# Experimental Investigation of Compression Properties of Composites with Printed Braiding Structure

**DOI:** 10.3390/ma11091767

**Published:** 2018-09-18

**Authors:** Zhengning Li, Ge Chen, Haichen Lyu, Frank Ko

**Affiliations:** 1 College of Mechanical Engineering, Donghua University, Shanghai 201620, China; 1149146@mail.dhu.edu.cn (Z.L.); llfllsllx@163.com (H.L.); 2 Department of Materials Engineering, University of British Columbia, Vancouver, BC V6T 1Z4, Canada; frank.ko@ubc.ca

**Keywords:** printed braiding structure, Epon 828, urethane dimethacrylate, triethylene glycol dimethacrylate, compression properties

## Abstract

A kind of composite was designed and additive manufacturing (AM) technology was utilized in the braiding structure fabrication. The printed polylactic acid (PLA) braiding structures were integrated with two types of resins (Epon 828 resin and urethane dimethacrylate/triethylene glycol dimethacrylate (UDMA/TEDGMA) resin) used as the matrix to make composite specimens. The compression test of the composite specimens showed that the printed PLA braiding structures had the effect of varying the compression properties of pure resins: it decreased the compression properties of Epon 828 resin, but increased those of UDMA/TEGDMA resin. Observing scanning electron microscope (SEM) images, it was noted that the decreasing and increasing in the compression properties of the specimens were related to the bonding compactness between the printed braiding structure and resins. Our results may suggest a new methods for the fast manufacturing of AM-based composites, further research directions, and potential applications of this kind of composites.

## 1. Introduction

Three dimensional (3D) braiding composite material has been widely applied in industrial areas for several decades, such as aeronautics and astronautics, as it has the advantages of a high modulus, high strength, excellent anti-delamination capability, and light weight [1,2,3]. According to the 3D braiding composite fabrication, it is common to use high-performance yarns to make the 3D braiding preform as the reinforcement first, and then introduce the resin into the 3D braiding preform as the matrix. Namely, the fabrication of 3D braiding composites consists of two steps: the 3D braiding preform fabrication and the resin integration. This method is time-consuming and costly, especially the step of 3D braiding preform fabrication, which substantially hinders the application of 3D braiding composite. 

**Figure 1:**
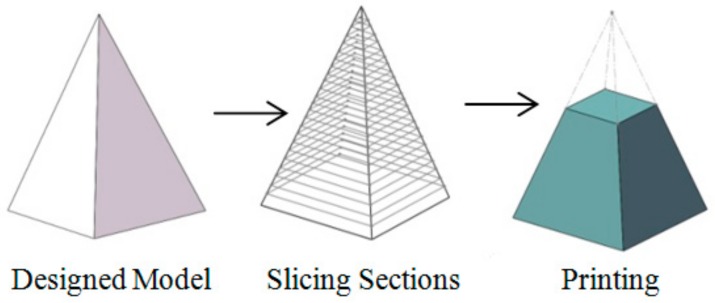
Schematic of 3D printing [8].

**Figure 2:**
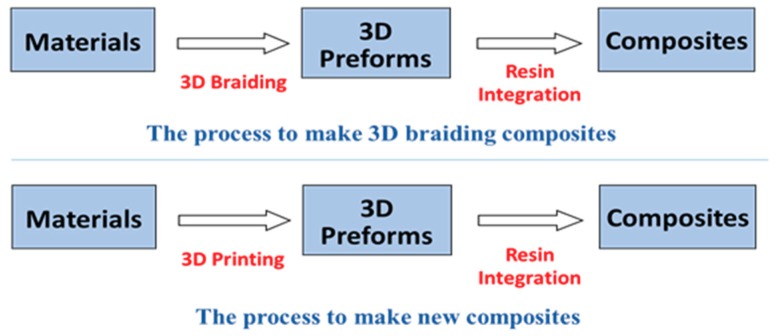
Comparison of classical and new processes to fabricate composites.

The development of additive manufacturing (AM) technology, also known as 3D printing, offers a brand-new method for composite designed and fabrication [4,5,6,7]. In the 3D printing process, the materials can be deposed layer by layer to make parts as long as the CAD model is imported into the 3D printer. The schematic of the 3D printing is shown in Figure 1 [8]. The model is designed in CAD software, and the control codes are then generated layer by layer in slicing software. Finally, the printer can print the parts according to control codes. This means that for the new kind of composite, the 3D braiding structure can be printed out directly and integrated with the resin. Thus, the preform design and fabrication process can be simplified greatly, while saving materials and time. The comparison of the traditional and new processes to fabricate composites is shown in Figure 2 [9,10,11]. However, the mechanical properties of composites with printed preform need further investigation in order to understand their potential applications. 

In the present work, we designed a process to fabricate composites using a printed 3D braiding structure as the reinforcement and resin as the matrix, and then performed a compression test on the specimens in the universal testing machine. Moreover, aiming to perform a micro-characterization of the fractured specimens, these were analyzed using a scanning electron microscope (SEM).

## 2. Materials and Methods

### 2.1. Printing of 3D Braiding Structure

**Figure 3:**
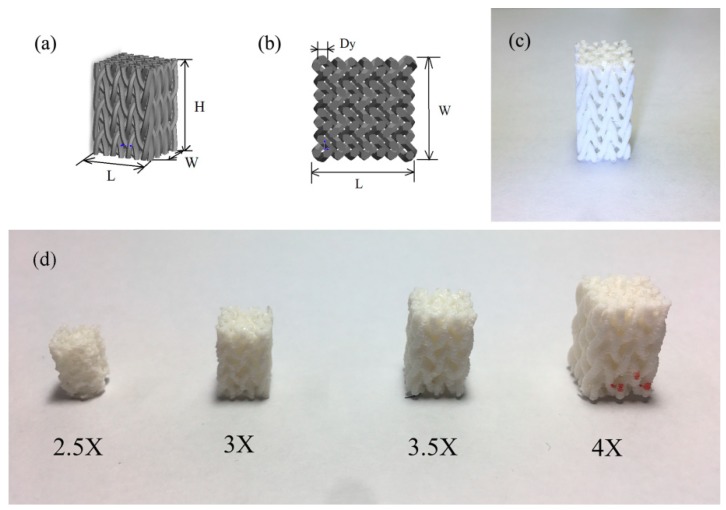
Braiding structure CAD model: (**a**) isometric view of braiding structure (L, W, H are the length, width, and height of the braiding structure); (**b**) top view of braiding structure (L, W are the length and width of the braiding structure, D_y_ is the diameter of the yarn in the braiding structure); (**c**) printed braiding structure with PLA filament; (**d)** experiment to ensure the fidelity of the printed specimen (from left to right: the yarn diameter in the braiding structure was amplified 2.5, 3, 3.5, and 4 times, respectively).

For the 3D printing work, a rectangular braiding structure model was first built in CAD software, as shown in Figure 3a,b, with a unit cell size of 0.87 mm. Moreover, the rectangular shape of the specimen resembled a tooth, which suggests its potential utilization in dentistry. The 3D printer for braiding structure manufacturing was Flashforge Creator (Flashforge Inc., Jinhua, China). The CAD model of the braiding structure needs to be saved in a stereolithography (STL) format and imported into slicing software to generate G-code to control the 3D printer. The thermal polymer material is deposited onto the bedplate to create printed models [12,13]. Considering the printing precision of the 3D printer and to ensure the fidelity of the printed model, the yarn diameter in CAD model needs to be amplified several times, such as 2.5, 3, 3.5, and 4 times. These four sizes of samples were printed to check the printing quality of models. In terms of the printing test, the diameter of yarns in the printed 3D braiding structure should be at least three times the nozzle diameter in order to guarantee printing fidelity, as shown in Figure 3d.

The rectangular braiding structure was printed according to the parameters in Table 1, as shown in Figure 3c. Regarding the printing speed, a speed below 80 mm per second was available for guaranteeing printing quality and a polylactid acid (PLA) filament was used to print the braiding structure. Because of the braiding structure’s repeated micro-structure, it was easy to create the required specimen sizes in pre-printing software. 

According to the standard test [14] method for the compressive properties of rigid plastics, the size of test specimen should be 12.7 mm (length) by 12.7 mm (width) by 25.4 mm (height). Considering the resin impregnation, the size of the printed braiding structure is slightly smaller than that of the test specimen; thus, the size of the printed braiding structure was 12.3 mm (length) by 12.3 mm (width) by 25.4 mm (height), as shown in Table 2.

### 2.2. Impregnation of Printed Braiding Structure with Resins 

**Figure 4:**
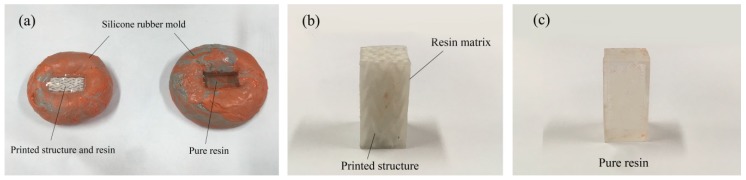
(**a**) Mold for printed braiding structure and resin impregnation. Left: printed braiding structure was put into the silicone rubber mold and resin was then infused into the mold; right: resin was infused into the silicone mold directly. Specimens for compression test: (**b**) specimen with printed braiding structures and resin matrix; (**c**) specimen of pure resin matrix.

In order to introduce the resin into the printed braiding structure, a rectangular mold was made with silicone rubber, as shown in Figure 4a, so that the printed braiding structure could be put into the mold and immersed in resin. Because of the splendid elasticity of the silicone rubber, after the resin has cured, the composite can be easily taken out of the mold. For the resin curing, the mold with the printed braiding structure and resin needs to be put into the vacuumed oven for 1 h to diminish the bubbles in the resin, or the resin may tend to crack during the curing process and the mechanical properties would be affected in the mechanical test. Subsequently, we adjusted the vacuum oven’s pressure to match the normal atmosphere and heated the sample to cure the resin. The parameters for resin curing are shown in Table 3. The resin needs to be cured under ordinary pressure; otherwise, it may crack and have bubbles in it after being cured.

For experimental comparison, two types of pure resin specimens were utilized, Epon 828 resin (Hexion Inc., Columbus, OH, USA) and dental resin (Bisco dental, Schaumburg, IL, USA). The dental resin was mixed with urethane dimethacrylate (UDMA) (Bisco dental, Schaumburg, IL, USA) and triethylene glycol dimethacrylate (TEGDMA) (Bisco dental, Schaumburg, IL, USA) with mixture ratio of UDMA/TEGDMA of 80/20 wt%; tert-butyl peroxybenzoate (Bisco dental, Schaumburg, IL, USA) was used as the initiator [15,16,17]. Epon 828 is a typical epoxy resin used for composite fabrication and the UDMA/TEGDMA resin is usually used in dentistry. Specimens of pure resins were also made and had the same size as the composite specimens. After removal from the silicone rubber molds, the pure resin specimens were polished in order to shape them as rectangular prisms, as shown in Figure 4b,c, and their size was 12.7 mm (length) by 12.7mm (width) by 25.4 mm (height). Following the definition of textile reinforcement composites, the printed braiding structure can be seen as reinforcement, and the resins can be seen as matrix. There were four types of specimens for compression test, as shown in Table 4.

### 2.3. Compression Test on Composite Specimens with Printed Braiding Structure

The universal testing system (Instron 3360, Instron Co., High Wycombe, UK) was utilized for the compression test. The compression velocity was 1 mm/min and stopped when specimens fractured. The fractured specimens were then coated with gold/palladium with a sputter coater (Denton Desk II, Denton Vacuum, Moorestown, NJ, USA) and observed with a scanning electron microscopy (SU8010, Hitachi High-tech Co., Tokyo, Japan) to characterize the cracked specimen surface [18,19,20].

## 3. Results and Discussion

### 3.1. Compression Properties of Different Specimens

**Figure 5:**
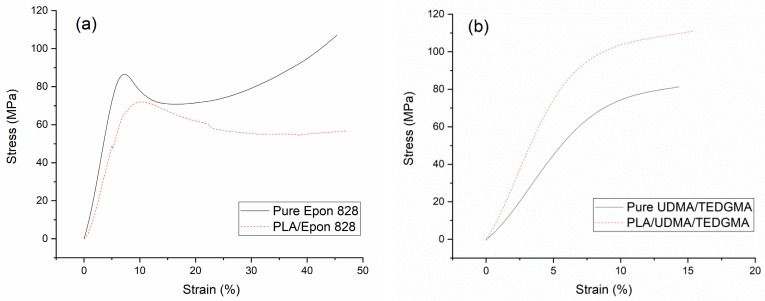
Compression stress-strain curves: (**a**) the solid line curve corresponds to the pure Epon 828 resin specimen (Type 1) and the short dash line curve to the PLA/Epon 828 specimen (Type 2); (**b**) the solid line curve corresponds to the UDMA/TEGDMA resin specimen (Type 3) and the short dash line curve to the PLA/ UDMA/TEGDMA specimen (Type 4).

Type 1 (pure Epon 828) specimen showed the typical stress-strain curves of ductile polymer materials; it yielded at about 4% strain and then had cold drawing until breaking. Type 2 (PLA/Epon 828) specimen, in which the Epon 828 was combined with the printed PLA braiding structure, yielded at about 8% strain, and both the yield stress and the breaking stress decreased, as shown in Figure 5a. Meanwhile, in the cold drawing section of Type 1 specimen the stress went down at first, and then went up gradually from 16% strain; on the contrary, in the cold drawing section of Type 2 specimen, the stress went down gradually until breaking. However, the whole stress-strain curve of Type 1 specimen was above that of Type 2 specimen. 

Type 3 (pure UDMA/TEGDMA resin) specimen did not have an obvious yield point until breaking, and broke at about 16% strain. Type 4 (PLA/UDMA/TEGDMA), in which the UDMA/TEGDMA resin was combined with the printed PLA structure, the breaking stress increased, as shown in Figure 5b. However, Type 3 and 4 specimens had very similar stress-strain trends, and thus did not show a difference as Type 1 (Pure Epon 828) and 2 (PLA/Epon 828) specimens did in the cold drawing sections. 

It was noticed that both Type 1 and 2 specimens yielded at less than 10% strain, and both Type 3 and Type 4 specimens broke at about 15% strain; thus, Type 2 tended to be a ductile polymer material due to the Epon 828 matrix. Correspondingly, Type 4 tended to be a semi-brittle polymer material, due to the UDMA/TEGDMA resin matrix.

**Figure 6:**
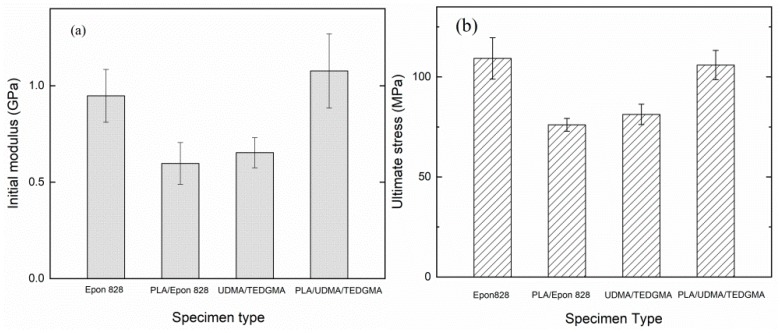
Mechanical properties of different specimens: (**a**) bar chart shows initial modulus of 4 types of specimens and their margins of error; (**b**) bar chart shows ultimate stress of 4 types of specimens and their margins of error.

Type 1 (Pure Epon 828) specimen had higher compressive properties than did Type 2 (PLA/Epon 828) specimen, such as initial modulus and ultimate stress, as shown in Figure 6a,b. Compared with Type 1 specimen, Type 2 specimen’s initial modulus went down by 37.03%, from 0.948 GPa to 0.597 GPa, and ultimate stress went down by 30.39%, from 109.226 MPa to 76.032 MPa. On the contrary, compared with Type 3 specimen, Type 4 specimen’s initial modulus and ultimate stress went up by 64.93% (from 0.653 GPa to 1.077 GPa) and 32.84% (from 81.246 MPa to 107.925 MPa), respectively. 

It seems that the PLA printed braiding structure cannot change the main trend of strain-stress curves of pure resins, but may change the compressive properties, such as initial modulus and ultimate stress. As shown in Figure 5a,b, the PLA printed braiding structure decreased the compressive properties of Epon 828 and improved the compressive properties of the UDMA/TEGDMA resin. 

In Reference [11], researchers explored the characterization of a printed 3D orthogonal structure with acrylonitrile-butadiene-styrene (ABS) and the compression properties of composites with silicone as matrix. They did not articulate the method of resin impregnation with the printed structure. Moreover, their target was to investigate the mechanical properties of the printed orthogonal structure, not the effect of the printed structure on different resins. According to the stress-strain curves of the specimens with and without the silicone matrix in Reference [11], the two stress-strain curves crossed at about 13% strain. However, for the composites we made, the stress-strain curves did not cross before fracture, as shown in Figure 5a,b. Furthermore, because the silicone was more ductile, the initial modulus of the composite specimen was only 0.12 GPa. It was thus noted that the composite specimens in the present work had more potential applications.

**Figure 7:**
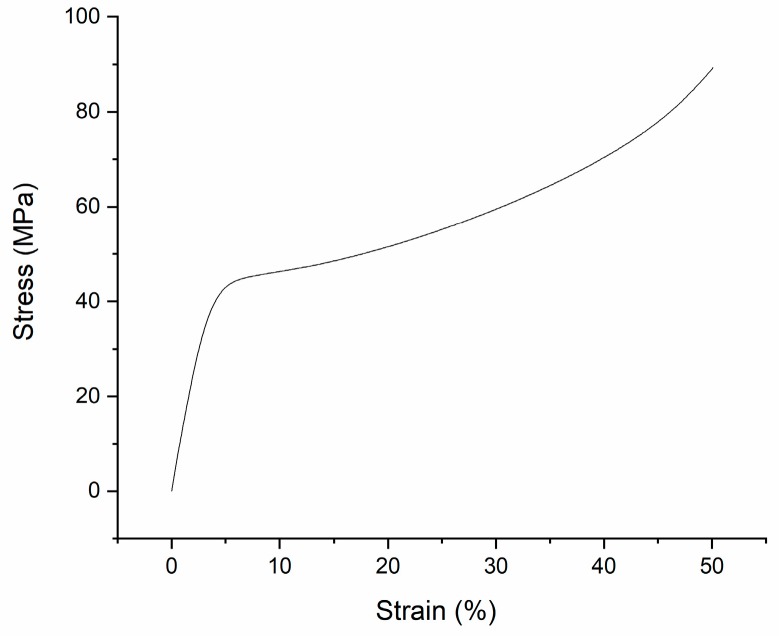
Compression stress-strain curve of solid PLA specimen.

In addition, a solid PLA specimen was printed to do the same compression test to compare with the composite specimens. The size of the solid PLA specimen was 13.7 mm (length) by 13.7 mm (width) by 25.4 mm (height). The compression strain-stress curve of the solid PLA specimen was shown in Figure 7. According to the compression strain-stress curve, the yield point of this specimen was at about 4% strain; subsequently, it seemed that the specimen’s stress went up in the cold drawing section but the solid PLA specimen actually fractured in this section, and the real ultimate stress of the solid PLA specimen was about 45 MPa. 

### 3.2. Specific Stiffness and Strength

In order to study the lightweight potential of composites, the specific stiffness and strength of composite specimens were calculated. 

The specimen’s density is defined as

(1) $$\rho =\frac{m}{V}$$

where V is the volume of the specimen, m is the mass of specimen.

Thus, the specific stiffness is

(2) $$\frac{E}{\rho }$$

where E is the compression initial modulus of the specimen, and ρ the density of specimen.

The specific strength is

(3) $$\frac{\sigma }{\rho }$$

where σ is the ultimate stress of the specimen.

All the variables above were calculated and are shown in Table 5.

According to the data in Table 5, the specimens with the printed PLA structures showed the same tendency in specific stiffness and strength as in initial modulus and ultimate strength. Compared with the pure Epon 828 specimen, the PLA/Epon 828 specimen’s specific stiffness decreased 28.02% and specific strength decreased 20.43%. Compared with pure UDMA/TEGDMA specimen, PLA/UDMA/TEGDMA specimen’s specific stiffness increased 74.41% and specific strength increased 40.61%. All in all, the PLA/UDMA/TEGDMA specimen had the best lightweight potential of all specimens.

### 3.3. Differential Scanning Calorimeter (DSC) Measurement for UDMA/TEGDMA Resin

**Figure 8:**
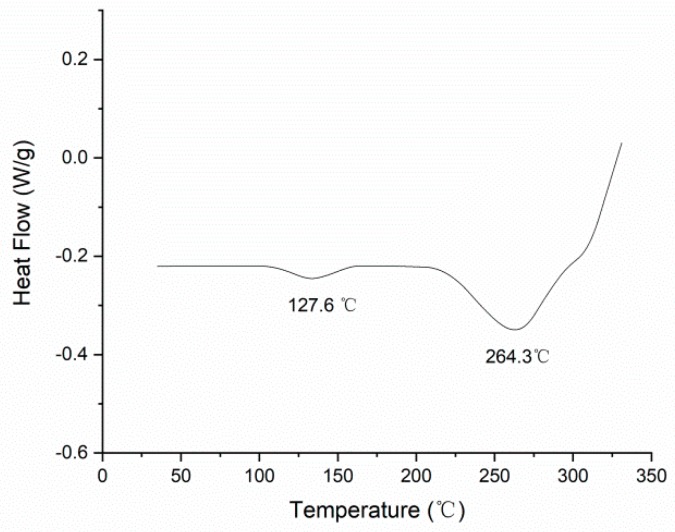
Differential scanning calorimeter (DSC) curve of UDMA/TEGDMA resin.

In order to test the curing conditions of the UDMA/TEGDMA resin and dismiss the effect of the resin’s uncompleted conversion, a differential scanning calorimeter (DSC) measurement was utilized. The DSC Q20 (TA instruments, New Castle, DE, USA) was used for this measurement, the increasing rate of temperature was 10 K per minute, and the specimen was the cured UDMA/TEGDMA resin. The DSC curve is shown in Figure 8.

According to the DSC curve, the glass transition temperature T_g_ of the UDMA/TEGDMA was about 127.6 °C and the melting point T_m_ about 264.3 °C. Commonly, the curing temperature should be lower than T_g_. Thus, the curing parameters we chose for the UDMA/TEGDMA resin were available to make the resin cured completely [21,22].

### 3.4. Characterization of Fractured Specimen Surfaces

**Figure 9:**
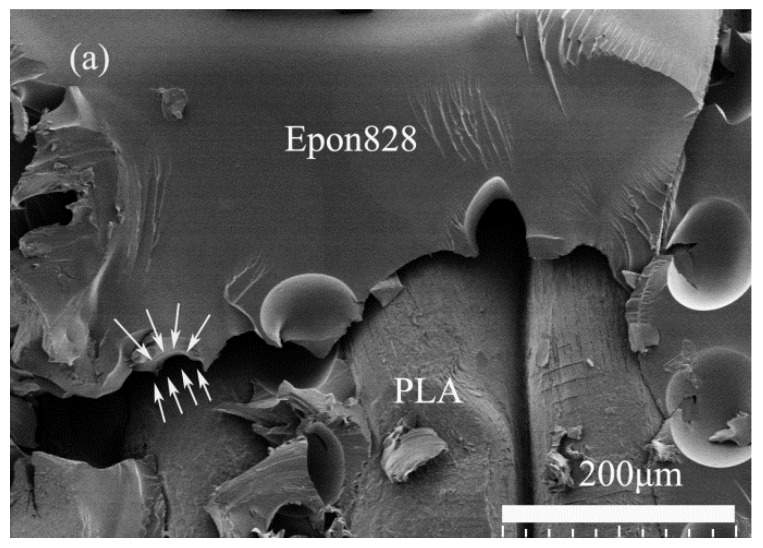

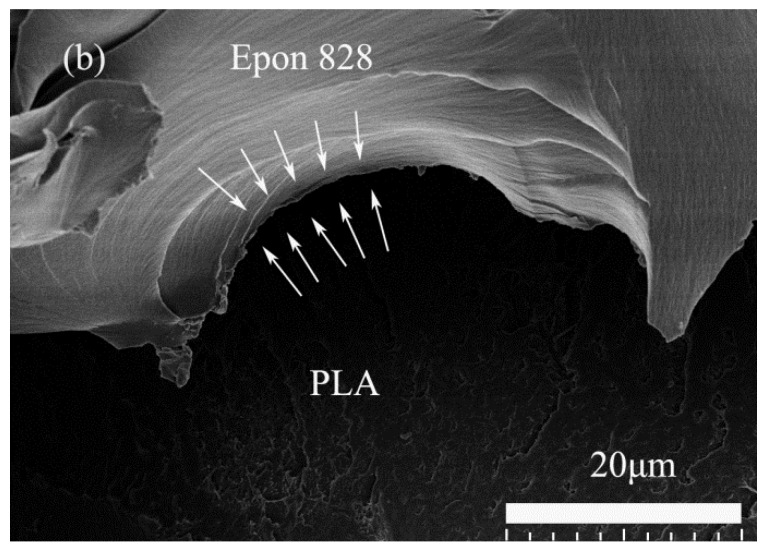
Scanning electron microscope (SEM) images of specimen’s fractured surface of Epon 828 resin and printed PLA braiding structure, with arrows pointing out the edge of these two materials: (**a**) scale of SEM image is 200 μm; (**b**) scale of SEM image is 20 μm.

In the PLA/Epon 828 system, the Epon 828 resin had a weak bonding with the printed PLA braiding structure, and these two materials separated from each other easily during the facture process. The boundary between these two materials was obvious, as shown in Figure 9a,b, because the printed PLA structure was nearly separated from the Epon 828 resin, and the fractured edge of the Epon 828 was sharp and had some small stripes along the fractured edge; thus, it is believed that in this system, the Epon 828 resin took the main part of fracture resistance. When adding the printed PLA braiding structure, the Epon 828 resin’s volume fraction decreased, which devalued both the initial modulus and ultimate stress of the pure Epon 828 resin specimen [23,24].

**Figure 10:**
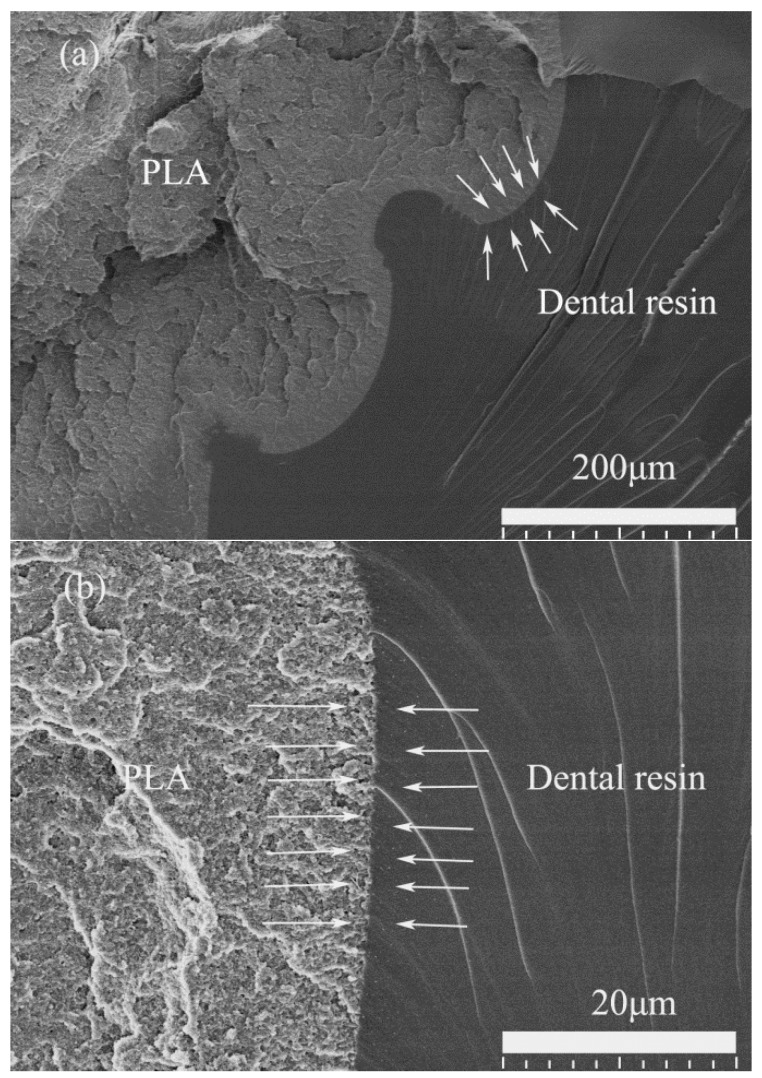
SEM images of specimen’s fractured surface of UDMA/TEGDMA resin and printed PLA braiding structure, with arrows pointing out the edge of these two materials: (**a**) scale of SEM image is 200 μm; (**b**) scale of SEM image is 20 μm.

Oppositely, in the PLA/UDMA/TEGDMA system, the UDMA/TEGDMA resin had strong bonding with the printed PLA braiding structure, and even though the specimen fractured, these two materials still cohered together at the fractured surface. Compared to the PLA/Epon 828 system, there is no obvious gap between the two materials, as shown in Figure 10a,b, and the small stripes on the UDMA/TEGDMA surface originated from the materials boundary, not along the boundary. Thus, it is believed that in this system, the printed PLA braiding structure enhanced the UDMA/TEGDMA resin’s compression properties because of the strong inter-phase bonding. 

**Figure 11:**
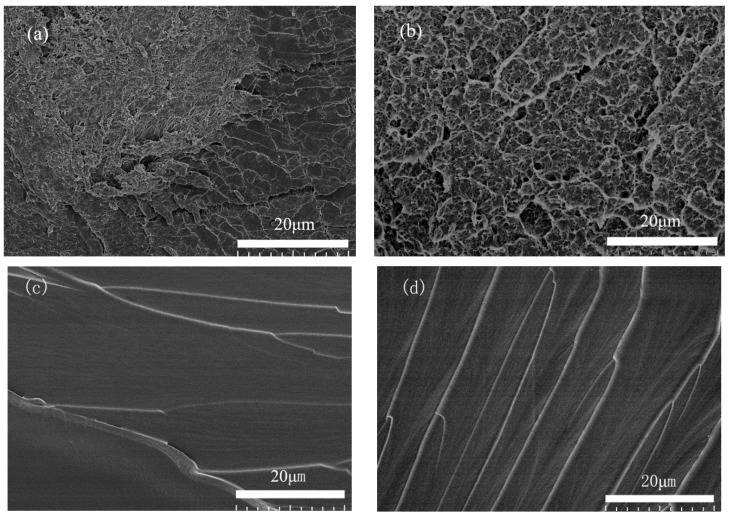
SEM images of (**a**) fractured PLA surface of Type 2 specimen; (**b**) fractured PLA surface of Type 4 specimen; (**c**) fractured Epon 828 resin surface of Type 2 specimen; (**d**) fractured UDMA/TEDGMA resin surface of Type 4 specimen.

According to these two types of SEM images of fractured specimens shown in Figure 11a,b, the fractured surfaces of PLA in both Type 2 (PLA/Epon 828) and Type 4 (UDMA/TEGDMA) specimens had a similar characterization, and were fully filled with pores due to the fused material deposition during the 3D printing process. As for the fractured resin surfaces of Type 2 and Type 4 specimens, they had flake patterns on the surfaces. In that case, it may be concluded that the printing quality of the PLA structure and the curing of the resins were good enough; thus, the variation in specimens’ compression properties had a direct relationship with the bonding compactness of the two materials, which are the printed reinforcement and resin matrix.

## 4. Conclusions

In this investigation, the aim was to verify the possibility to fabricate composites of printed structures with a resin matrix, and to then study the compression properties of the composites. 

The experiments showed that the printed PLA braiding structure changed the resin’s compression properties, such as initial modulus and ultimate stress, but could not change the trend of strain-stress curves. Moreover, the change in compression properties may be related to the bonding compactness between the materials. For example, because of the light bonding between the printed PLA braiding structure and Epon 828 resin, Type 2 (PLA/Epon 828) specimen’s initial modulus and ultimate stress decreased by 37.03% and 30.39% respectively, compared to pure Epon 828 resin specimen; furthermore, given the strong bonding between the printed PLA braiding structure and UDMA/TEGDMA resin, Type 4 (PLA/UDMA/TEGDMA) specimen’s initial modulus and ultimate stress increased by 64.93% and 32.84% respectively, compared to pure UDMA/TEGDMA resin. This suggests that the printed braiding structures could be used to enhance the compression properties of resins and may offer a convenient method for composite fabrication. Moreover, the PLA/UDMA/TEDGMA specimen showed the best lightweight potential among all specimens. 

This study provides some insights for future research: (1) other materials can be tried to print braiding structures in order to check the compression properties, such as acrylonitrile-butadiene-styrene (ABS) and nylon; (2) the braiding angle of CAD models can be changed to check the variation in the printed structure’s volume fraction; (3) other printed structures can be infused with resin to make composites, such as honeycomb, woven, and knitting structures. Through these studies, the application of this new kind of composites may be expanded. 

## Figures and Tables

**Table 1:** Parameters of four-step braiding structure printing.

Parameter	Value
Nozzle diameter	400 µm
Layer thickness	150 µm
Nozzle temperature	210 °C
Bed temperature	90 °C
Printing speed	30 mm/s
Filament materials	Polylactid acid (PLA)

**Table 2:** Parameters of printed braiding structure.

Parameter	Value
Length	12.3 mm
Width	12.3 mm
Height	25.4 mm
Yarn diameter	1.05 mm

**Table 3:** Resin curing parameters.

Resin Type	Curing Temperature	Curing Time
Epon828	50 °C	12 h
Urethane dimethacrylate/triethylene glycol dimethacrylate	100 °C	1 h

**Table 4:** Specimen types and materials.

Specimen Type	Materials of Printed Braiding Structure	Resin Matrix
1	-	Epon 828
2	PLA	Epon 828
3	-	UDMA/TEGDMA
4	PLA	UDMA/TEGDMA

**Table 5:** Density, specific stiffness, and specific strength of different specimens.

Specimen Type	Density (kg/m^3^)	Specific Stiffness (10^6^ m^2^/s^2^)	Specific Strength (kN·m/kg)
Solid PLA	999.74	1.20	45.01
Epon 828	1144.87	8.28	95.40
PLA/Epon 828	1001.55	5.96	75.91
UDMA/TEDGMA	1413.97	5.51	68.49
PLA/UDMA/TEGDMA	1120.68	9.61	96.30

## References

[1] Bilisik K. (2013). Three-dimensional braiding for composites: A review. Text. Res. J..

[2] Chou T.W., Ko F.K. (1989). Textile Structural Composites.

[3] Chou T.W. (2005). Microstructural Design of Fiber Composites.

[4] Hufenbach W., Böhm R., Thieme M., Winkler A., Mäder E., Rausch J., Schade M. (2011). Polypropylene/glass fibre 3D-textile reinforced composites for automotive applications. Mater. Des..

[5] Hull C.W. (1986). Apparatus for Production of Three-Dimensional Objects by Stereolithography.

[6] American Society for Testing Materials (2012). Standard Terminology for Additive Manufacturing Technologies.

[7] Mitschang P., Blinzler M., Wöginger A. (2003). Processing technologies for continuous fiber reinforced thermoplastics with novel polymer blends. Compos. Sci. Technol..

[8] Quan Z., Wu A., Keefe M., Qin X., Yu J., Suhr J., Byun J., Kim B., Chou T. (2015). Additive manufacturing of multi-directional preforms for composites: Opportunities and challenges. Mater. Today.

[9] Huang Y., Leu M.C., Mazumder J., Donmez A. (2015). Additive manufacturing: Current state, future potential, gaps and needs, and recommendations. J. Manuf. Sci. Eng..

[10] Ahuja B., Karg M., Schmidt M. Additive manufacturing in production: Challenges and opportunities. Proceedings of the SPIE LASE.

[11] Quan Z., Larimore Z., Wu A., Yu J., Qin X., Mirotznik M., Suhr J., Byun J., Oh Y., Chou T. (2016). Microstructural design and additive manufacturing and characterization of 3D orthogonal short carbonfiber/acrylonitrile-butadiene-styrene preform and composite. Compos. Sci. Tech..

[12] Petrovic V., Vicente J., Jordá F. (2011). Additive layered manufacturing: Sectors of industrial application shown through case studies. Int. J. Prod. Res..

[13] Ding D., Pan Z., Cuiuri D., Li H. (2014). A tool-path generation strategy for wire and arc additive manufacturing. Int J. Adv. Manuf. Tech..

[14] American Society for Testing Materials (2010). Standard Test Method for Compressive Properties of Rigid Plastics.

[15] McCrum N.G., Buckley C.P., Bucknall C.B. (1997). Principles of Polymer Engineering.

[16] Finger W.J., Fritz U.B. (1997). Resin bonding to enamel and dentin with one–component UDMA/HEMA adhesives. Eur. J. Oral Sci..

[17] Asmussen E., Peutzfeldt A. (1998). Influence of UEDMA, BisGMA and TEGDMA on selected mechanical properties of experimental resin composites. Dent. Mater..

[18] Perez A.R.T., Roberson D.A., Wicker R.B. (2014). Fracture surface analysis of 3D-printed tensile specimens of novel ABS-based materials. J. Fail. Anal. Prev..

[19] Li C., Liang R., Ren J. (2016). Comparative study on friction properties of different dental restorative materials against natural tooth enamel and dentin. Chin. J. Mater. Res..

[20] Kim J., Baillie C., Poh J., Mai Y.W. (1992). Fracture toughness of CFRP with modified epoxy resin matrices. Compos. Sci. Technol..

[21] Santana I.L., Gonçalves L.M., Ribeiro J.J.S., Mochel Filho J.R., Júnior C., Alves A. (2011). Thermal behavior of direct resin composites: Glass transition temperature and initial degradation analyses. Rev. Odonto Ciênc..

[22] Hayakawa T., Takahashi K., Kikutake K., Yokota I., Nemoto K. (1999). Analysis of polymerization behavior of dental dimethacrylate monomers by differential scanning calorimetry. J. Oral Sci..

[23] Jordan W.M., Bradley W.L., Moulton R.J. (1989). Relating resin mechanical properties to composite delamination fracture toughness. J. Compos. Mater..

[24] Gresnigt M.M., Özcan M., van den Houten M.L., Schipper L., Cune M.S. (2016). Fracture strength, failure type and Weibull characteristics of lithium disilicate and multiphase resin composite endocrowns under axial and lateral forces. Dent. Mater..

